# Herb-Induced Liver Injury: A Report on Two Australian Cases

**DOI:** 10.7759/cureus.24686

**Published:** 2022-05-03

**Authors:** Varun Peri, Catherine Yu, Karl Vaz, Khashayar Asadi, Josephine Grace

**Affiliations:** 1 Surgery, Austin Health, Melbourne, AUS; 2 General Medicine, Austin Health, Melbourne, AUS; 3 Gastroenterology and Hepatology, Austin Health, Melbourne, AUS; 4 Pathology, Austin Health, Melbourne, AUS

**Keywords:** complementary and alternative medicine(cam), alternative medicine, supplements, drug induced liver injury, hepatitis, herb induced liver injury

## Abstract

Two 40-year-old males were admitted to our tertiary hepatology unit with acute hepatitis after presentation with generalized abdominal pain, nausea, and jaundice. There was no history of paracetamol overdose, and common viral and autoimmune causes were excluded through serology. Imaging and liver biopsy were performed with both investigations demonstrating non-specific features of hepatic inflammation. A history of herbal supplement use was elucidated in each patient, which was deemed to be the cause of liver injury in both men. Each patient recovered within two months of presentation following the withdrawal of the offending agent and supportive care.

## Introduction

Herb-induced liver injury is an uncommon cause of acute hepatitis in patients admitted to hospital, with myriad complementary and alternative medicines (CAM) implicated [1]. Given CAM use is prevalent in over 60% of the Australian population, patient education and raising awareness of the potentially severe consequences of these often benign supplements are critical in reducing the incidence of morbidity and mortality [2,3]. The relative rarity of these presentations and lack of definitive diagnostic tests often leads to a prolonged and costly workup and delay in identifying the underlying etiology of liver injury.

## Case presentation

We present two cases (patients A and B) with herb-induced liver injury. Both patients were Caucasian males aged 40-years who were admitted to the hospital following two weeks of generalized cramping abdominal pain, nausea without vomiting, jaundice, and pruritus. Neither patient reported fevers or any recent risk factors for the acquisition of viral hepatitis. Neither patient had a history of mental health disorders, and both reported a stable mood with no suspicion of deliberate self-poisoning. Patient A volunteered a history of excess alcohol consumption (~ 60g/day), with abstinence since the onset of icteric illness, and an L4-radiculopathy secondary to a herniated disc requiring, on average, 3 gm of paracetamol per week with the last dose of 1 gm administered three days before presentation. Patient B had a seizure disorder secondary to a traumatic brain injury that was controlled on levetiracetam with prior normal liver function tests (LFT) monitored by his general practitioner. Both patients noted the use of oral supplements purchased over the internet. Patient A was taking corydalis for over 18 months for its purported analgesic properties, with the last ingestion one week before admission. Patient B used a multi-herbal supplement that contained, amongst other components, Tongkat ali root (*Eurycoma longifolia*), horny goat weed, and saw palmetto fruit extract, for the presumed anabolic effect on skeletal muscle.

The clinical examination of both patients was unremarkable except for jaundice. Both were apyrexic and had no peripheral stigmata of chronic liver disease, metabolic flap, or organomegaly. Initial biochemical testing showed significant LFT derangement in each patient, with differing patterns. Patient A had a predominant transaminitis on admission with evidence of synthetic and excretory hepatic dysfunction. The full blood examination (FBE), and urea, electrolytes, creatinine (UEC) were normal. Patient B had a predominantly cholestatic pattern of liver enzymatic derangement. Apart from a mild thrombocytosis, the remainder of the FBE was normal and there was no evidence of kidney injury or electrolyte derangement. Viral serology for hepatitis A, B, C, and E, and herpes simplex virus, Epstein-Barr virus, cytomegalovirus, and parvovirus were negative in both patients. Circulating markers of autoimmune hepatitis (anti-nuclear, anti-smooth muscle, anti-liver/kidney/microsomal-1 antibodies, and total immunoglobulin G level) were not raised in either patient, and paracetamol levels were below the level of detection in patient A. Hyperferritinemia was present in patient A (5,199 ug/L) but not patient B (229 ug/L) and was attributed to hepatic inflammation. Patient A had sonographic evidence of hepatitis with homogenously hypoechoic liver parenchyma, and Doppler examination revealed patent portal and hepatic vasculature. Patient B had normal hepatic parenchyma and biliary system on magnetic resonance cholangiopancreatography. Histopathological examination of liver biopsy in patient A revealed severe acute lobular hepatitis without confluent necrosis or fibrosis, nor features in keeping with alcoholic hepatitis, autoimmune hepatitis, or metabolic causes of liver injury and patient B had a biopsy that showed acute cholestasis with mild cholangiopathy, consistent with drug-induced cholestatic injury.

The herbal supplements were ceased in both patients, and they were admitted for diagnostic workup and supportive care. Patient A was hospitalized for four days, and patient B for five days. Both experienced rapid resolution of pruritus and abdominal pain early in their admission and were asymptomatic on discharge, barring jaundice. Complete biochemical recovery was observed two months post-admission in both patients (Table 1), with the only remaining abnormality being a mildly elevated gamma-glutamyl transferase (GGT) in patient A likely reflecting ongoing alcohol consumption, and a minimally elevated alkaline phosphatase (ALP) in patient B. They were advised not to re-challenge with the implicated supplements given the severity of the presentation.

**Table 1:** LFT and platelet values of Patient A and Patient B at different time points of their admission LFT: Liver function tests, OP: Outpatient, ALT: Alanine aminotransferase, AST: Aspartate aminotransferase, ALP: Alkaline phosphatase, GGT: Gamma-glutamyl transferase, INR: International normalized ratio

Parameter (reference range)	Admission	Discharge	OP clinic (1 month)	OP clinic (2 months)
ALT (5-40 units/L)				
Patient A	3732	4348	275	33
Patient B	69	55	126	39
AST (< 40 units/L)				
Patient A	1782	2072	52	Not available
Patient B	50	64	85	Not available
GGT (< 60 units/L)				
Patient A	476	440	115	37
Patient B	39	3	44	19
ALP (30-110 units/L)				
Patient A	190	191	131	80
Patient B	318	318	238	113
INR				
Patient A	1.7	1.4	1.2	1.1
Patient B	1.1	1.2	1.0	1.0
Bilirubin (< 21 μmol/L)				
Patient A	109	133	18	11
Patient B	182	241	100	16
Albumin (35-50 g/L)				
Patient A	38	36	39	44
Patient B	31	26	31	41
Platelets (150-400 x 10^9^)				
Patient A	343	405	321	352
Patient B	451	455	Not available	355

## Discussion

Drug-induced liver injuries (DILI) are an uncommon cause of acute liver failure in Western countries, with a heterogeneous clinical course from complete resolution with the withdrawal of the offending agent to fulminant liver failure necessitating emergent transplantation [1]. They are challenging to diagnose, requiring a high index of suspicion, meticulous investigation for alternate etiologies that frequently leads to liver biopsy with its inherent risks, and often necessitate an expert consensus on the probability that the implicated agent is the most likely cause of injury.

Complementary and alternative medicine (CAM) use in Australia has consistently been demonstrated to be over 60% in surveys of the general population [2,3]. Complementary and alternative medicine tend not to be subject to the same rigorous clinical trials of safety and efficacy as allopathic medicines and can equally evade scrutiny from pharmacovigilance studies. As such, information regarding their adverse effect profile is often under-reported or unknown. This, coupled with ease of access given they do not require a prescription from a medical practitioner leads to a common misconception that their use is entirely safe.

Corydalis is a species of herbal plant, the extract of which is used for its putative analgesic properties. Although rarely reported in the literature, it is a known cause of herb-induced liver injury [4,5]. The case we describe is, to the best of our knowledge, the first reported case of corydalis-induced liver injury in Australia. It is unclear which component of the mixed herbal supplement was the hepatotoxic agent for patient B, as Tongkat ali root, horny goat weed, and saw palmetto fruit extract have all been implicated in causing clinically significant liver injury [6,7]. This difficulty in ascertaining the specific cause of liver injury with CAMs is often encountered in clinical practice, as CAMs are infrequently marketed as single ingredients and are more commonly combined with several potentially hepatotoxic ingredients.

Drug-induced liver injuries are thought to occur via several mechanisms, including direct toxic effect or production of metabolites leading to impairment or alteration of the structural or functional or integrity of the liver, systemic hypersensitivity reaction and immune-mediated liver damage [8-10]. Histological findings in these patients differ based on the mechanism of hepatic injury. The hepatitic reaction pattern is often similar to that of viral infection or immune-mediated hepatitis, and a raised autoantibody (particularly anti-nuclear and anti-smooth muscle antibodies) can also be present, in this case requiring correlation with other laboratory parameters and histology. Varying cholestatic injury patterns can be induced by herbal supplements from bland bilirubin stasis to sclerosing cholangitis [11] or vanishing bile duct syndrome [12]. 

## Conclusions

The two cases of herb-induced liver injury highlight an uncommon cause of acute hepatitis in patients presenting to a tertiary hepatology unit. Given the absence of definitive laboratory investigations that can support this diagnosis, a detailed exposure history is important to obtain at the time of the first review. Especially as a missed diagnosis and subsequent failure to cease the offending agent can lead to potentially morbid consequences. 
